# Mental health among Chinese university students during COVID-19: 28-month, ten-wave longitudinal study

**DOI:** 10.1192/bjo.2024.869

**Published:** 2025-03-20

**Authors:** Wendy Wen Li, Daniel Miller, Christopher Rouen, Fang Yang, Huizhen Yu

**Affiliations:** 1 College of Healthcare Sciences, James Cook University, Townsville, Queensland, Australia; 2 Department of Social Work, Foshan University, Foshan, China

**Keywords:** Anxiety, COVID-19 lockdown, depression, longitudinal, mental health

## Abstract

**Background:**

The cumulative effects of long-term exposure to pandemic-related stressors and the severity of social restrictions may have been important determinants of mental distress in the time of COVID-19.

**Aim:**

This study aimed to investigate mental health among a cohort of Chinese university students over a 28-month period, focusing on the effects of lockdown type.

**Methods:**

Depression, anxiety, stress and fear of COVID-19 infection were measured ten times among 188 Chinese students (females 77.7%, mean_age_ = 19.8, s.d._age_ = 0.97), every 3 months: from prior to the emergence of COVID-19 in November 2019 (T1) to March 2022 (T10).

**Results:**

Initially depression, anxiety and stress dipped from T1 to T2, followed by a sudden increase at T3 and a slow upward rise over the remainder of the study period (T3 to T10). When locked down at university, participants showed greater mental distress compared with both home lockdown (*d* = 0.35–0.48) and a no-lockdown comparison period (*d* = 0.28–0.40). Conversely, home lockdown was associated with less anxiety and stress (*d* = 0.19 and 0.21, respectively), but not with depression (*d* = 0.13) compared with a no-lockdown period.

**Conclusions:**

This study highlights the cumulative effects of exposure to COVID-19 stressors over time. It also suggests that the way in which a lockdown is carried out can impact the well-being of those involved. Some forms of lockdown appear to pose a greater threat to mental health than others.

Coronavirus disease 19 (COVID-19) was first identified in December 2019 and was declared a pandemic by the World Health Organization in March 2020.^1^ Governments around the world quickly enacted social restrictions and quarantine measures to mitigate the spread of the virus. This created concerns about the impact that these measures, and of other pandemic-related stressors (e.g. fear of infection), might have on mental health,^2–4^ spurring research on the topic.

Available evidence suggests that the pandemic negatively impacted mental health. One recent meta-analysis of longitudinal cohort studies reports significant increases in depression and anxiety symptoms during the early pandemic when compared with the prepandemic period (with this increase being higher for depression than anxiety) in countries outside of China.^5^ However, among studies of Chinese participants, no significant increase in mental health symptoms was observed. The authors also report that changes in mental health symptoms from the prepandemic period diminished with each passing month. However, the majority of studies included in this review completed data collection during the early stages of the pandemic and thus these findings cannot address the cumulative effects of long-term exposure to COVID-19-related stressors. The review’s authors call for researchers to continue to monitor mental health as the pandemic continued.

Although there were more than 60 longitudinal studies into university students’ mental health during COVID-19, the large majority of longitudinal studies completed data collection in 2020, with only two collecting data into 2021.^6,7^ Barbieri et al^6^ report a 34% increase (relative to prepandemic levels) in the symptoms of depression 1 year into the pandemic among a sample of US university students; depression levels had not returned to prepandemic levels by the conclusion of the study (April 2021). Yaghi^7^ similarly found that participants (Jordanian undergraduates studying public affairs) continued to display greater symptoms of stress and anxiety into early 2021.

Regarding the effects of social restrictions specifically, one meta-analysis suggests that social restrictions are associated with increases in depression symptoms and stress, and loneliness, but not with anxiety symptoms.^8^ Strictness and length of social restrictions were found to impact mental health, but in divergent ways (e.g. stricter restrictions were associated with greater depression but less anxiety). These authors note that many of the included studies make global assumptions about participants’ experiences with social restrictions (e.g. based on what was happening at the national level within the country in which data were collected). Nonetheless, the findings appear to suggest that imposed social restrictions can be detrimental to mental health, and that the stringency of social restrictions influences this impact.

One particularly stringent form of social restriction is lockdown. Lockdown has been defined as ‘a set of measures aimed at reducing transmission of COVID-19 that are mandatory, applied indiscriminately to a general population and involve some restrictions on the established pattern of social and economic life’^9^ (p. 2), and may include home or geographical confinement. Research from Australia indicates that strict lockdown is associated with poorer mental health among parents and children, as compared with less stringent social restrictions.^10^

The present paper responds to the need to ‘ascertain the effects of lockdown and social isolation over time’^4^ (p. 551) through the study of a cohort of Chinese university students (previously reported in Li et al^11^) measured at ten time points over a 28-month period (November 2019 to March 2022). The study investigates mental health outcomes (symptoms of depression, anxiety and stress), as well as fear of infection, among this cohort of university students, with a particular focus on the effects of lockdown type on mental health. The homogeneity of the group under study means that we can have more certainty regarding participants’ experiences with social restrictions as compared with prior research.^8^ To the authors’ knowledge, the present study is the only longitudinal COVID-19 study to extend data collection into 2022 and collect data during the spread of the main COVID-19 variants (e.g. the original COVID-19 virus and the Delta and Omicron variants).

## Method

### Participants and study design

The authors originally planned to collect data at three time points only (reported in Li et al^11^). When planning for the original study, a priori sample size analysis was conducted (in G*Power v.3.1.9.4). This analysis determined that a sample size of 163 would result in a power of 0.80 to detect a change in an outcome variable over three time points, assuming a small effect size (*f* = 0.10), large correlations between time point measures (*r* = 0.50) and an *α* of 0.05. It was assumed that correlations between repeated measures would be large, based on the test–retest reliability of the scale used in the current study.^12^ Thus, we aimed to recruit around 200 participants to account for likely attrition.

Following the emergence of COVID-19, the data collection period was extended to capitalise on the opportunity to investigate the longer-term impacts of COVID-related stressors on students’ mental health. The period of data collection was extended to reflect the duration required for the majority of participants to complete their studies (because maintaining communication with participants following graduation would probably prove challenging). Given that data collection was later extended to ten time points, sensitivity analysis was also conducted following data collection to determine the smallest effect size that could be detected with a power of 0.80. This analysis indicated that, based on the achieved sample size (*N* = 188), number of measurements,^10^ average correlation between repeated measures (mean correlation between time point measures was 0.53, 0.51 and 0.51 for depression, anxiety and stress, respectively) and an *α* of 0.05, repeated-measures analysis of variance (ANOVA) could detect a small-sized (*f* = 0.06) main effect with a power of 0.80. Thus, the analysis is underpowered to detect any main effect lower than *f* = 0.06.

Convenience sampling was used to recruit students from the School of Public Administration and Management at Foshan University, China. This school incorporates the disciplines of social work, international economics, economics and trade, marketing and accounting. Emails inviting participants to take part in a longitudinal study into the mental health of university students were sent to all students in these disciplines (*N* = 619). No exclusion criteria were applied – that is, all invited participants were eligible to participate.

A total of 203 students completed the survey battery in November 2019 (T1), prior to the COVID-19 pandemic. Participants who participated in at least eight time points were included in the current analysis (*N* = 188). This cohort consisted of 146 females (77.7%) and 42 males (22.3%), with an average age of 19.8 years (range = 18–22, s.d. = 0.97) at T1. There were 46 (24.5%), 25 (13.3%), 24 (12.8%), 48 (25.5%) and 45 (23.9%) participants in the disciplines of social work, international economics, economics and trade, marketing and accounting, respectively.

All procedures contributing to this work complied with the ethical standards of the relevant national and institutional committees on human experimentation, and with the Helsinki Declaration of 1975 as revised in 2013. All procedures involving human participants were approved by the Human Research Ethics Committee (HREC) of the Department of Social Work, Foshan University (Ref. 2019001) and the HREC of James Cook University (Ref. H8214).

Written informed consent was obtained from all participants. A paper-and-pencil survey was administrated at T1, while online surveys were conducted via https://www.wjx.cn/ from T2 to T10. Each participant received RMB ¥8.88 from T3 to T10 (remuneration was provided following participation at each time point). Data were collected approximately every 3 months; Table 1 presents details of each study time point.

**Table 1:** Study time points, with academic and COVID-19 stressors noted

Time point	Month	Academic period	COVID-19 stressors
T1	Nov 2019	Midsemester exam period	Prior to onset of COVID-19
T2	Feb/Mar 2020	End of winter holiday/start of the new semester	Pandemic lockdown, students in home lockdown
T3	May/Jun 2020	Between midsemester and final exam periods	University closed, online learning from home
T4	Aug 2020	End of the summer holiday	Students returned to campus, facemasks mandated, social distancing
T5	Nov 2020	Midsemester exam period	Students on campus, F-T-F teaching, face masks mandated, social distancing
T6	Feb 2021	End of winter holiday	Students returned to campus, face masks mandated, social distancing, start of vaccine rollout.
T7	Jun 2021	Between midsemester and final exam periods	Students on campus, students in university lockdown to prevent spread of Delta variant, face masks mandated in accommodation, online learning in accommodation
T8	Aug 2021	Start of semester	Students on campus, F-T-F teaching, face masks mandated, social distancing
T9	Nov 2021	Midsemester exam period	Students on campus, F-T-F teaching, face masks mandated, social distancing
T10	Feb/Mar 2022	End of winter holiday/start of new semester	Students returned to campus, F-T-F teaching, face masks mandated, social distancing, massive PCR testing due to spread of Omicron variant

F-T-F, face to face; PCR, polymerase chain reaction.

Midsemester examinations generally account for 40% of overall grade.

At T10, some cities in China entered city lockdown to prevent spread of the Omicron variant.

During the 28-month study period, participants were twice measured while under COVID-19 lockdown – in February/March 2020 (T2) and June 2021 (T7). However, the nature of these lockdowns was different. During the first lockdown period (T2), the university was temporarily closed and all participants resided in their familial homes during the period (hereafter referred to as home lockdown). The students’ university remained open during the second lockdown period (T7), with participants engaging in lockdown from their dormitories (hereafter referred to as university lockdown). While residing in their dormitories, participants lived with three to four other students in a 30–40 m^2^ room.

### Measures

This study focuses on three mental health-related outcome variables (frequency and severity of symptoms of depression, anxiety and stress) and fear of COVID-19 infection. Demographic variables were also assessed (age, sex and discipline of study). Symptoms of depression, anxiety and stress were measured using the standardised Chinese version of the 21-item Depression Anxiety Stress Scale (C-DASS-21).^13^ C-DASS-21 includes seven items each for depression, anxiety and stress, using a four-point scale (where 0 denotes ‘did not apply to me at all’ and 3 denotes ‘applied to me very much, or most of the time’). As suggested by the authors of this scale, subscale scores were multiplied by two (thus outcome scores range from 0 to 42, with higher scores indicating greater depression, anxiety and stress) to be consistent with the 42-item DASS-42.^13^ Although DASS-42 and its derivative scales take a dimensional rather than categorical approach to psychological disorders, Lovibond and Lovibond^14^ report severity cut-off values for DASS-42. These were applied to determine the percentage of participants with elevated (mild to extremely severe) levels of depression, anxiety and stress at each time point (reported in Table 2). It should, however, be noted that these severity cut-off values were derived from non-clinical samples of Australians and are not specific to Chinese populations. C-DASS-21 has demonstrated good internal consistency in recent studies.^11,15,16^ Cronbach’s *α* values for the depression, anxiety and stress subscales in the current study ranged 0.84–0.91, 0.73–0.91 and 0.78–0.92 respectively, across the ten time points.

**Table 2:** Mean values (s.d.) for depression, anxiety, stress and fear of infection (FOI); percentage of participants with elevated levels of depression, anxiety and stress; and Pearson’s correlations between fear of infection and depression, anxiety and stress

	Depression			Anxiety			Stress			FOI
Time point	Mean (s.d.)	Percentage elevated	Correlation with FOI	Mean (s.d.)	Percentage elevated	Correlation with FOI	Mean (s.d.)	Percentage elevated	Correlation with FOI	Mean (s.d.)
T1	6.21 (6.28)	25.10	–	9.50 (6.36)	61.00	–	11.23 (7.21)	30.90	–	–
T2	5.08 (6.15)	20.80	0.01	5.21 (5.95)	31.50	0.04	7.21 (7.20)	16.90	0.02	2.10 (0.91)
T3	6.34 (6.26)	28.70	0.21**	7.15 (6.50)	42.00	0.29***	9.21 (7.31)	22.90	0.25***	2.18 (1.05)
T4	5.95 (6.63)	23.90	0.23**	7.03 (6.63)	42.00	0.29***	9.38 (8.13)	26.10	0.34***	2.00 (0.98)
T5	6.39 (6.41)	31.90	0.29***	7.32 (6.62)	46.30	0.34***	9.85 (7.74)	25.00	0.36***	1.97 (1.00)
T6	6.72 (7.34)	31.90	0.21**	7.59 (7.10)	45.70	0.20**	9.82 (8.50)	27.70	0.26***	1.94 (0.93)
T7	7.18 (7.12)	35.10	0.18*	8.44 (7.20)	48.90	0.17*	10.76 (8.07)	27.70	0.23**	2.02 (0.94)
T8	7.77 (7.70)	37.40	0.13	8.92 (7.55)	52.40	0.15*	11.21 (8.50)	31.60	0.13	1.93 (0.88)
T9	7.24 (7.29)	35.80	0.17*	9.02 (8.27)	52.40	0.22**	10.53 (8.68)	28.90	0.29***	1.89 (0.97)
T10	7.64 (7.27)	37.60	0.14	9.01 (7.91)	50.80	0.19*	11.10 (8.35)	30.90	0.21**	1.95 (1.00)

Number of completed responses: T1, 187; T2, 178; T3–T7, 188; T8–T9, 187; and T10, 181. FOI not measured at T1. *r* values represent the correlation between depression, anxiety or stress and FOI within each timepoint (e.g. correlation of T2 depression with T2 FOI). Elevated depression, anxiety and stress were determined based on severity cut-off values provided by Lovibond and Lovibond.^14^ All participants who met mild, moderate, severe or extremely severe severity thresholds were categorised as ‘elevated’.

*P < 0.05, **P < 0.01, ***P < 0.001.

Fear of COVID-19 infection was measured by two questions adapted from the Health Anxiety Inventory.^17^ The measure was administered across nine time points (fear of infection was not measured at T1, because this was prior to the onset of COVID-19). Respondents were asked to rate how frequently they worried about themselves and their parents contracting COVID-19, on a four-point scale (where 0 denotes ‘not at all’ and 3 denotes ‘very often’), with outcome scores ranging from 0 to 6 (where higher scores indicate greater fear of COVID-19 infection). Cronbach’s *α* for this scale was 0.64 at T2, but ranged from 0.72 to 0.84 for the remainder of the study period.

### Statistical analysis

Data (available online)^18^ were analysed using IBM SPSS Statistics (v.28) and R (v.4.0.3). As a preliminary analytical step, data were assessed for missing data points and univariate outliers. Outside of those who entirely skipped a time point, missing data were minimal, with almost all scale items having no missing responses and two missing one response only; expectation maximisation was used to estimate values for these two missing data points. Total scores were assessed for univariate outliers using the outlier labelling rule with a 2.2 multiplier.^19^ To retain as many data as possible, outlying data points were assigned the next-highest observed, non-outlying value +1 (to maintain the rank order of data). In total, 16 data points were replaced following this method.

Pearson’s correlations were used to investigate the association between fear of infection and mental health outcomes at each time point, with coefficient values of 0.10, 0.30 and 0.50 being considered representative of small, medium and large relationships, respectively.^20^

Repeated-measures ANOVA was used to assess whether mental health outcomes and fear of infection changed over time. It was determined that significant ANOVA would not be followed up with a comparison of all time points via post hoc testing (because there are 45 unique pairwise comparisons that could be made for each outcome variable). Rather, significant ANOVA was followed up with targeted paired *t*-tests focusing on changes in mental health/fear of infection in response to different public health measures. Specifically, this analysis involved comparisons of outcome variables during the lockdown periods – T2 (home lockdown) and T7 (university lockdown) – with these being compared both against each other and against non-lockdown periods at the same point in the academic year (to control for the effects of university-related stressors, such as examinations). These comparisons are made for the overall sample and male and female subsamples. To control type 1 error rate, an adjusted *α* level of 0.0125 (0.05 divided by the number of outcome variables) was applied to these *t*-tests. Standardised differences between means were computed via Cohen’s *d*, with 0.20, 0.50 and 0.80 being considered as representative of small, medium and large differences, respectively.^20^

Graphs were also produced to demonstrate patterns of change in the outcome variables over the study period. Decorrelated confidence intervals (CIs) – adjusted using the Cousineau–Morey technique (implemented via the ‘superb’ package in R)^21^ – are presented in these plots. In order that CI overlap heuristics^22^ can be applied, CIs presented in the figures have been ‘decorrelated’; decorrelated CIs are adjusted to account for positive correlations between time point measures in repeated-measures designs. These CIs were calculated based on those who participated at all time points (*N* = 169–170). CIs were not, what Cousineau et al^21^ describe as, ‘difference adjusted’.

## Results

### Association between mental health outcomes and fear of infection at each time point

Pearson’s correlations between fear of infection and depression, anxiety and stress are provided in Table 2, along with means and standard deviations at each time point. As indicated in Table 2, T2 fear of infection was not associated with either T2 depression, anxiety or stress. From T3 to T7, and again at T9, fear of infection positively correlated with depression, anxiety and stress (a medium-sized relationship in most cases).

### Changes in mental health outcomes and fear of infection over time

Repeated-measures ANOVAs indicated significant changes in depression over the study period (*N* = 169, *F*[6.56, 1102.02] = 5.10, *P* < 0.001, partial *η* ^2^ = 0.03), anxiety (*N* = 169, *F*[6.82, 1145.31] = 12.18, *P* < 0.001, partial *η* ^2^ = 0.07), stress (*N* = 170, *F*[6.88, 1164.29] = 9.37, *P* < 0.001, partial *η* ^2^ = 0.05) and fear of infection (*N* = 170, *F*[6.62, 1118.67] = 2.08, *P* < 0.046, partial *η* ^2^ = 0.01). For all four ANOVAs, degrees of freedom were corrected using Huynh–Feldt estimates of sphericity, given violations of the assumption of sphericity.

**Fig. 1:**
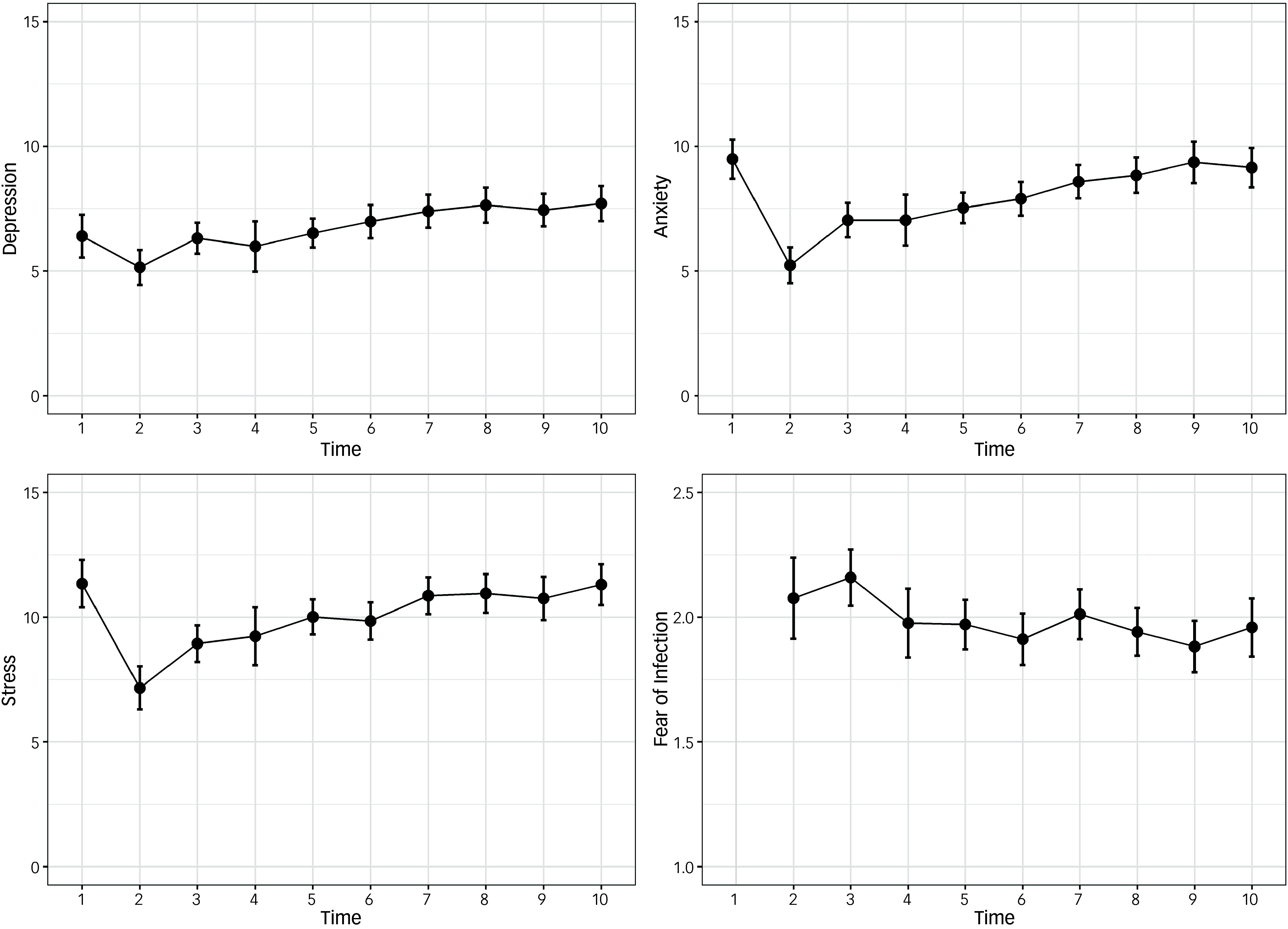
Mean depression, anxiety stress and fear of infection (with decorrelated 95% CIs), from November 2019 (T1) to February/March 2022 (T10), among the overall sample.

**Fig. 2:**
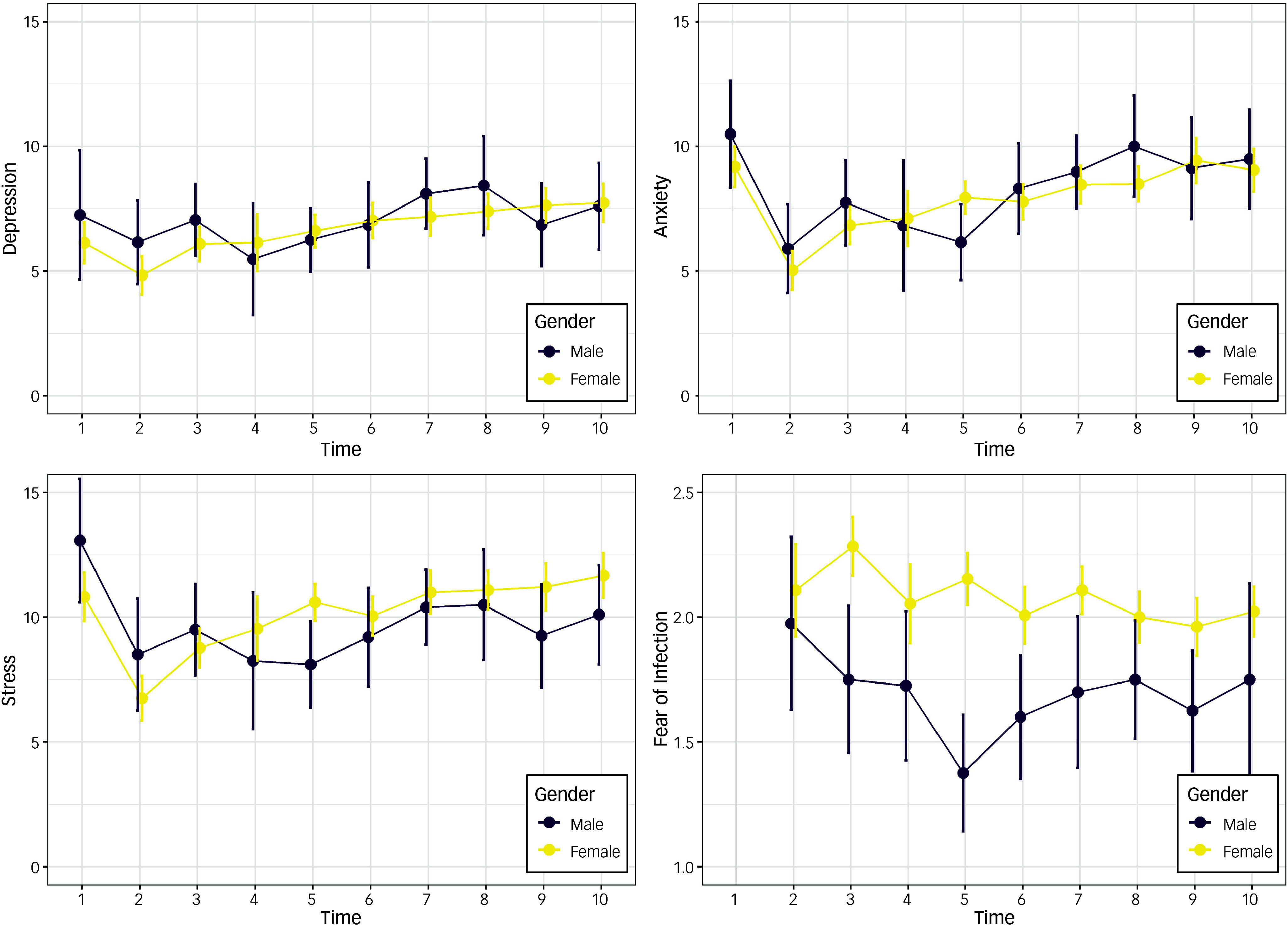
Mean depression, anxiety stress and fear of infection (with decorrelated 95% CIs), from November 2019 (T1) to February/March 2022 (T10) among males (*N* = 42) and females (*N* = 146).

Independent sample *t*-tests indicated no significant differences between males and females in regard to depression, anxiety or stress at any time point (except for T1 stress, with males displaying greater stress; see S1–S3 in Supplementary Materials available at https://doi.org/10.1192/bjo.2024.869). Patterns of change in outcome variables are depicted in Figs 1 and 2.

In relation to fear of infection, the highest levels were observed at the beginning of the pandemic (T2 and T3), after which fear of infection decreased. Increases were observed at T7 and T10. These time points correspond to the spread of the Delta and Omicron variants in China, respectively.^23,24^ Independent sample *t*-tests confirmed significant gender differences at T3 through to T9 for fear of infection, with males displaying significantly less fear of infection in all cases (see S4 in Supplementary Materials).

### Changes in mental health outcomes and fear of infection during lockdown

Participants reported significantly greater depression, anxiety and stress during university lockdown (T7) compared with home lockdown (T2), with small-to-medium and medium differences being observed (see Table 3). This pattern of findings was mirrored in the female subsample. No differences were observed for the male subsample (if applying an *α* of 0.0125). It should be noted that these tests are underpowered due to the small number of males in the sample. Sensitivity analysis indicates an ability to detect effects as low as *d* = 0.52 and *d* = 0.27 with a power of 0.80 among male and female subsamples, respectively.

**Table 3:** Comparison of depression, anxiety, stress and fear of infection during (1) home lockdown (T2) and university lockdown (T7), (2) home lockdown (T2) and a no-lockdown period 1 year later (T6) and (3) a no-lockdown period (T3) and university lockdown 1 year later (T7)

Variable	T2 (Mean (s.d.))	T7 (Mean (s.d.))	Mean difference	*t*	*d.f.*	*P*	Cohen’s *d*
Overall sample							
Depression	5.08 (6.15)	7.28 (7.19)	−2.11	−4.39	177	<0.001	0.35
Anxiety	5.21 (5.95)	8.42 (7.24)	−3.20	−6.39	177	<0.001	0.48
Stress	7.21 (7.20)	10.76 (8.11)	−3.55	−5.98	177	<0.001	0.45
Fear of infection	2.10 (0.91)	2.00 (0.95)	0.10	0.99	177	0.324	0.07
Males							
Depression	6.00 (6.72)	7.90 (8.22)	−1.90	−1.83	40	0.074	0.29
Anxiety	5.71 (6.35)	8.68 (8.12)	−2.98	−2.60	40	0.013	0.41
Stress	8.29 (8.12)	10.15 (8.40)	−1.85	−1.45	40	0.154	0.23
Fear of infection	2.00 (0.97)	1.66 (1.20)	0.34	1.40	40	0.168	0.22
Females							
Depression	4.81 (5.97)	7.09 (6.87)	−2.28	−4.32	136	<0.001	0.37
Anxiety	5.07 (5.84)	8.34 (6.98)	−3.27	−5.88	136	<0.001	0.50
Stress	6.89 (6.90)	10.95 (8.04)	−4.06	−6.10	136	<0.001	0.52
Fear of infection	2.12 (0.89)	2.10 (0.84)	0.02	0.22	136	0.830	0.02

Variable	T2 (Mean (s.d.))	T6 (Mean (s.d.))	Mean difference	*t*	*d.f.*	*P*	Cohen’s *d*
Overall sample							
Depression	5.08 (6.15)	6.83 (7.37)	−1.75	−3.75	177	<0.001	0.28
Anxiety	5.21 (5.94)	7.74 (7.18)	−2.52	−5.33	177	<0.001	0.40
Stress	7.21 (7.21)	9.81 (8.51)	−2.60	−4.52	177	<0.001	0.34
Fear of infection	2.10 (0.91)	1.92 (0.93)	0.18	1.78	177	0.076	0.13
Males							
Depression	6.00 (6.72)	6.68 (8.18)	−0.49	−0.61	40	0.546	0.10
Anxiety	5.71 (6.35)	7.90 (8.39)	−2.20	−1.96	40	0.057	0.31
Stress	8.29 (8.12)	8.98 (9.10)	−0.68	−0.51	40	0.612	0.08
Fear of infection	2.00 (0.97)	1.56 (1.00)	0.44	2.10	40	0.043	0.33
Females							
Depression	4.81 (5.97)	6.88 (7.14)	−2.07	−4.10	136	<0.001	0.35
Anxiety	5.07 (5.84)	7.69 (6.80)	−2.62	−5.06	136	<0.001	0.43
Stress	6.89 (6.90)	10.06 (8.34)	−3.17	−5.07	136	<0.001	0.43
Fear of infection	2.12 (0.89)	2.02 (0.88)	0.10	0.89	136	0.374	0.08

Variable	T3 (Mean (s.d.))	T7 (Mean (s.d.))	Mean difference	*t*	*d.f.*	*P*	Cohen’s *d*
Overall sample							
Depression	6.34 (6.26)	7.18 (7.11)	−0.85	−1.78	187	0.076	0.13
Anxiety	7.15 (6.50)	8.44 (7.20)	−1.29	−2.63	187	0.009	0.19
Stress	9.21 (7.31)	10.76 (8.07)	−1.54	−2.81	187	0.006	0.21
Fear of infection	2.18 (1.05)	2.02 (0.94)	0.17	2.27	187	0.024	0.17
Males							
Depression	6.95 (5.96)	7.81 (8.14)	−0.86	−0.82	41	0.420	0.13
Anxiety	7.52 (7.11)	8.67 (8.02)	−1.14	−1.05	41	0.299	0.16
Stress	9.38 (7.10)	10.10 (8.30)	−0.71	−0.61	41	0.547	0.09
Fear of infection	1.71 (1.17)	1.67 (1.18)	0.05	0.26	41	0.800	0.04
Females							
Depression	6.16 (6.36)	7.00 (6.82)	−0.84	−1.58	145	0.116	0.13
Anxiety	7.04 (6.34)	8.37 (6.97)	−1.33	−2.42	145	0.017	0.20
Stress	9.16 (7.39)	10.95 (8.02)	−1.78	−2.86	145	0.005	0.24
Fear of infection	2.32 (0.97)	2.12 (0.83)	0.20	2.58	145	0.011	0.21

Number of completed responses: T1, 187; T2, 178; T3–T7, 188; T8–T9, 187; and T10, 181.

d.f., degree of freedom.

Participants experienced significantly less depression, anxiety and stress during home lockdown (T2) as compared with the same period 1 year later (T6), with small-to-medium differences being observed (see Table 3). This pattern of results also applied to the female subsample. Among males, depression, anxiety and stress did not significantly differ between the two periods.

Participants experienced significantly more anxiety and stress, but not depression, during university lockdown (T7) as compared with the same period 1 year earlier (T3), with small differences being observed (see Table 3). The female subsample displayed significantly greater stress during university lockdown, but not greater depression or anxiety (if applying an *α* of 0.0125). Once again, no differences were observed for the male subsample.

Among the overall sample, fear of infection did not differ between T2 and T6, T3 and T7 or T2 and T7 (if applying an adjusted *α* level of 0.0125). This pattern of findings was the same among the male subsample. Among the female subsample, fear of infection was found to be greater during the T3 no-lockdown period compared with university lockdown (a small effect).

## Discussion

### Changes in mental health outcomes and fear of infection over time

This study sought to investigate mental distress in Chinese university students over a 28-month period, with a particular focus on distress during COVID-19 lockdowns. In the cases of depression, anxiety and stress, an initial dip in mental distress moving from the pre-COVID to COVID-19 period (T1 to T2) was observed, before a sudden increase at T3 – as described in our previous publication^11^ – and a slow upward trend over the remainder of the study period (T3 to T10). This general trajectory was observed among both males and females.

The trajectories for depression, anxiety and stress are consistent with Zunin-Myers’ Phases of Disaster model, which proposes that, following major disasters, people may experience a heroic and honeymoon phase during which heroic stories emerge (e.g. frontline healthcare workers battling the COVID-19 pandemic), community bonding occurs and optimism prevails.^25^ During these phases, people are optimistic that life will soon return to normal. Lower levels of depression, anxiety and stress at T2 may reflect that participants were then in the heroic and honeymoon stages of disaster recovery. Following these phases, optimism often turns to disillusionment when stress, fatigue and worry begin to affect mental health.^25^ The slow increase in severity of stress, anxiety and depression symptoms that was observed from T2 to T10 may reflect a transition out of the honeymoon period, as participants became increasingly aware of the pandemic’s impact on mortality both nationwide and globally. Another possibility is that at T2, most participants were residing in geographical areas with low levels of infection and few confirmed cases. From T5 onwards, participants had returned to campus and thus may have been living in a geographic region with more confirmed cases. Li et al^15^ found that mental distress is positively associated with the level of infection severity in one’s area of residence. Alternatively, the sudden drop in distress at T2 may reflect events external to the pandemic. For example, T1 and T2 fell during the midsemester examination period and at the end of winter holiday period, respectively. High levels of mental distress are common among students during examination periods, whereas holiday periods are often associated with less distress.^26^ However, it should be noted that multiple other time points coincided with the end of holiday periods but were not associated with the same sudden drop in mental distress.

Previous research indicates that female university students tend to have a higher prevalence of mental disorders compared with their male counterparts.^27^ Consequently, one might expect that male participants would have scored significantly lower than females on the outcome variables at most time points in the study. However, no significant differences were observed between males and females for stress, anxiety or depression across any of the ten time points (with the exception of T1, for which males actually showed higher levels of stress). It is also worth noting that male participants may have been inclined to underreport their true levels of distress, given that aspects of Chinese culture promote emotional control as a masculine norm.^28^

One possible explanation for these unexpected findings around gender is that male university students are more reactive to certain pandemic-related stressors, resulting in their mental distress being elevated to a level comparable to that of their female counterparts. This would suggest that special consideration should be given to the mental health of male university students during public health crises. However, it is also true that depression, anxiety and stress symptoms did not differ in response to lockdown among the male subsample, which runs counter to this differential reactivity hypothesis.

Fear of infection was greatest 3 months into the pandemic (T3), followed by periods when the COVID-19 Delta and Omicron variants emerged in China. This trend suggests that fear of infection appears to be associated with the level of infection severity in one’s residential area.

### Positive association between fear of COVID-19 infection and mental health outcomes

The observed positive correlations between fear of infection and mental health suggest that worry about oneself or one’s family contracting COVID-19 was one of the drivers of mental distress in the time of COVID-19. Perceived vulnerability to COVID-19 infection has been found to be associated with stronger emotional reactions to COVID-related threats and greater anxiety.^29^ That said, the correlations observed in the present study (Table 2) were typically modest in size, which could also suggest that fear of infection is not the sole, or even primary, driver of mental distress among this population. It is possible that this is because the sample comprises university students who may perceive themselves to be less personally susceptible to infection, or less likely to become seriously ill if infected, given their age.^11,30^

### Changes in mental health outcomes and fear of infection during lockdown

The results of this study suggest that lockdown type is an important determinant of mental distress. Participants displayed significantly fewer symptoms of depression, anxiety and stress during home lockdown relative to both university lockdown and the no-lockdown comparison period. Conversely, university lockdown was associated with significantly greater depression, anxiety and stress relative to both home lockdown and the no-lockdown comparison period. Meta-analytic evidence suggests that stricter social restrictions are associated with less anxiety, but greater depression.^8^ This was not observed in the current study, where more stringent lockdown measures (T7 university-based lockdown) were associated with worse mental health uniformly across depression, anxiety and stress.

Interestingly, fear of infection was not found to differ between lockdown types or between lockdown periods and no-lockdown comparison periods (with the exception of female participants displaying greater fear of infection during the no-lockdown comparison period relative to university lockdown). This would suggest that the observed decreases and increases in mental distress during home and university lockdown, respectively, cannot be attributed to participants feeling any less or more fearful about COVID-19 infection. Possible reasons for reductions in mental distress during home lockdown include increased familial support and greater flexibility in schedules. More flexible schedules may have allowed participants opportunities to engage in novelty-seeking behaviours, such as creative pursuits or new hobbies (Li et al^11^). Lower levels of mental distress at T2 lockdown, as compared with T7 lockdown, may also be attributable to participants being in the honeymoon period of disaster recovery (mentioned above) at T2, during which time lockdown might have been perceived as ‘new’ and ‘exciting’.

### Limitations and strengths

Although the gender ratio observed in the current sample is reflective of that of the five participating disciplines at the university targeted for recruitment, the relatively small subsample of males impeded our ability to conduct analyses separately by gender or compare genders against each other. Non-significant findings in relation to gender should be interpreted with these power limitations in mind. Moreover, the findings of this study are limited to university students (and, in particular, Chinese university students studying social work- and business-orientated degrees) and cannot be generalised to the whole population, or even to all university students.

Due to the large number of time point measures, consideration should also be given to the possibility of panel conditioning – a phenomenon in which participants’ response behaviours when completing a survey are influenced by virtue of these having previously been measured repeatedly.^31^ Panel conditioning can occur when participants recall their previous responses and attempt to maintain consistency at later time points. It can also result from participants being sensitised to the study’s topic (in this case, one’s experiences of mental distress), prompting them to reflect on the topic more frequently in their daily lives and potentially altering their experiences. However, we note that DASS and its derivative measures explicitly instruct participants to respond in relation to their feelings over the previous week, which may discourage any expectation of consistency. Additionally, the relatively long period between time points (3 months) probably also reduces the impact of memory effects. Despite this, participant sensitisation to the study topic remains a possibility.

Despite these limitations, this study possesses a number of strengths. First, it has a long data collection period relative to other longitudinal studies in this area,^8^ facilitating more detailed insight into the longer-term effects of the pandemic on the mental health of the study population. Second, the relatively homogenous sample allows for greater certainty as to what social restrictions were being imposed on participants (as opposed to applying global assumptions based on national-level information).^8^ Third, the current study had a relatively low attrition rate, despite ten waves of data collection being conducted over an extended period.

### Conclusion and implications

This research extends previous longitudinal studies by examining changes over a 28-month period prior to, and during, the COVID-19 pandemic and investigating associations between fear of infection and mental health during the pandemic. To the authors’ knowledge, this is the first study to highlight trajectories of mental health over 2 years into the COVID-19 pandemic, and the impact of the restrictions imposed during lockdown on mental health outcomes and fear of infection. The current study observed an initial dip in levels of depression, anxiety and stress at the beginning of the pandemic, followed by a sudden increase after 3 months and a slow upward trend thereafter. It was found that fear of infection was positively associated with mental distress at most time points. Experiencing lockdown at university was associated with significantly greater mental distress compared with home lockdown or unrestricted periods.

Available evidence indicates that the COVID-19 pandemic had a negative impact on mental health globally. The findings of the current study offer several implications to policy-makers and practising mental health professionals. First, it provides important information on the potential psychological impacts of lockdown type on university students. This information can assist in informing public health decision-making when designing and implementing lockdown measures during infectious disease events, and has important clinical implications. Second, the current study found that, after an initial dip in mental distress at the beginning of the COVID-19 pandemic, there was an upward trend in distress over the remainder of the study period, with changes also being observed across lockdown types. This finding delivers an important message: the impact of the pandemic on the public’s mental health is not static, but instead appears to change as a function of both time since the start of the pandemic and the stringency of public health measures (e.g. more severe lockdown types). The changing trajectories of mental health during the pandemic should be considered in the design and implementation of mental health programmes and treatments in response to COVID-19 and future pandemics. Third, this study reports a positive association between fear of infections and mental health outcomes. This finding provides mental health professionals with empirical evidence for the need to develop programmes (such as mindfulness intervention)^32^ to help people manage their fear of being infected by COVID-19 or other future pandemic diseases when addressing mental health issues.

## Supporting information

Li et al. supplementary material Li et al. supplementary material

## Data Availability

The data that support the findings of this study are openly available in online repositories: The Research Data JCU (https://doi.org/10.25903/6hrt-qg88).

## Supplementary material

The supplementary material is available online at https://doi.org/10.1192/bjo.2024.869
