# Supplementary material for: Mental health among Chinese university students during COVID-19: 28-month, ten-wave longitudinal study
Source: BJPsych Open. 2025 Mar 20;11(2):e56. doi: 10.1192/bjo.2024.869 (PMC12001943; doi:10.1192/bjo.2024.869)
Supplement: Li et al. supplementary material [file S205647242400869Xsup001.docx]

Supplementary Material To:

**Mental Health before and during the COVID-19 Pandemic among Chinese Late Adolescents and Emerging Adults: A 28-Month, 10-Wave Longitudinal Study**

**Table S1**

*Independent Samples t tests comparing male (n = 42) and female (n = 146) participants on depression at each time point.*

|  | Mean (SD) | |  |  |  |  |  |
| --- | --- | --- | --- | --- | --- | --- | --- |
| Depression | **Males** | **Females** | **Mean difference** | ***t*** | ***df*** | ***p*** | ***d*** |
| T1 | 7·62 (8·46) | 5·92 (6·03) | 1·46 | 1·46 | 185 | ·146 | 0·26 |
| T2 | 6·20 (7·35) | 4·86 (6·15) | 1·16 | 1·16 | 176 | ·247 | 0·21 |
| T3 | 6·95 (5·96) | 6·19 (6·46) | 0·68 | 0·68 | 186 | ·495 | 0·12 |
| T4 | 6·00 (7·24) | 6·08 (6·89) | -0·07 | -0·07 | 186 | ·946 | -0·01 |
| T5 | 6·10 (5·84) | 6·48 (6·58) | -0·34 | -0·34 | 186 | ·733 | -0·06 |
| T6 | 6·57 (8·11) | 6·77 (7·14) | -0·20 | -0·15 | 186 | ·880 | -0·03 |
| T7 | 7·81 (8·14) | 7·00 (6·82) | 0·81 | 0·65 | 186 | ·518 | 0·11 |
| T8 | 9·33 (9·99) | 7·37 (7·09) | 1·97 | 1·43 | 185 | ·153 | 0·25 |
| T9 | 6·90 (8·52) | 7·34 (6·92) | -0·43 | -0·34 | 185 | ·736 | -0·06 |
| T10 | 7·66 (7·57) | 7·63 (7·20) | 0·03 | 0·02 | 179 | ·982 | > 0·01 |
|  |  |  |  |  |  |  |  |

**Table S2**

*Independent Samples t tests comparing male (n = 42) and female (n = 146) participants on anxiety at each time point*

|  | Mean (SD) | |  |  |  |  |  |
| --- | --- | --- | --- | --- | --- | --- | --- |
| Anxiety | **Males** | **Females** | **Mean difference** | ***t*** | ***df*** | ***p*** | ***d*** |
| T1 | 11·12 (8·06) | 9·15 (6·19) | 1·97 | 1·68 | 185 | ·095 | 0·30 |
| T2 | 5·71 (6·35) | 5·07 (5·84) | 0·64 | 0·61 | 176 | ·546 | 0·11 |
| T3 | 7·52 (7·11) | 7·04 (6·34) | 0·48 | 0·42 | 186 | ·673 | 0·07 |
| T4 | 6·86 (7·33) | 7·08 (6·44) | -0·23 | -0·19 | 186 | ·847 | -0·03 |
| T5 | 5·76 (5·90) | 7·82 (6·94) | -2·06 | -1·75 | 186 | ·082 | -0·31 |
| T6 | 7·76 (8·34) | 7·64 (7·14) | 0·12 | 0·09 | 186 | ·928 | 0·02 |
| T7 | 8·67 (8·02) | 8·37 (6·97) | 0·30 | 0·23 | 186 | ·815 | 0·04 |
| T8 | 10·29 (9·37) | 8·52 (6·92) | 1·76 | 1·33 | 185 | ·184 | 0·23 |
| T9 | 8·90 (9·68) | 9·05 (7·85) | -0·14 | -0·10 | 185 | ·921 | -0·02 |
| T10 | 9·22 (8·32) | 8·94 (7·81) | 0·28 | 0·20 | 179 | ·844 | 0·03 |
|  |  |  |  |  |  |  |  |

**Table S3**

*Independent Samples t tests comparing male (n = 42) and female (n = 146) participants on stress at each time point.*

|  | Mean (SD) | |  |  |  |  |  |
| --- | --- | --- | --- | --- | --- | --- | --- |
| Stress | **Males** | **Females** | **Mean difference** | ***t*** | ***df*** | ***p*** | ***d*** |
| T1 | 13·38 (9·05) | 10·67 (6·74) | 2·71 | 2·12 | 186 | ·036 | 0·37 |
| T2 | 8·29 (8·12) | 6·89 (6·90) | 1·40 | 1·09 | 176 | ·275 | 0·19 |
| T3 | 9·38 (7·10) | 9·16 (7·39) | 0·22 | 0·17 | 186 | ·866 | 0·03 |
| T4 | 8·71 (8·52) | 9·58 (8·03) | -0·86 | -0·60 | 186 | ·547 | -0·11 |
| T5 | 7·95 (7·09) | 10·40 (7·86) | -2·44 | -1·81 | 186 | ·071 | -0·32 |
| T6 | 8·95 (8·99) | 10·07 (8·37) | -1·12 | -0·75 | 186 | ·455 | -0·13 |
| T7 | 10·10 (8·30) | 10·95 (8·02) | -0·85 | -0·60 | 186 | ·549 | -0·11 |
| T8 | 11·19 (9·64) | 11·21 (8·17) | -0·02 | -0·02 | 185 | ·988 | > ·01 |
| T9 | 9·29 (9·13) | 10·90 (8·55) | -1·61 | -1·06 | 185 | ·291 | -0·19 |
| T10 | 10·10 (8·01) | 11·40 (8·46) | -1·30 | -0·88 | 179 | ·381 | -0·16 |
|  |  |  |  |  |  |  |  |

**Table S4**

*Independent Samples t tests comparing male (n = 42) and female (n = 146) participants on fear of infection at each time point*

|  | Mean (SD) | |  |  |  |  |  |
| --- | --- | --- | --- | --- | --- | --- | --- |
| Fear of Infection | **Males** | **Females** | **Mean difference** | ***t*** | ***df*** | ***p*** | ***d*** |
| T1 | - | - | - | - | - | - | - |
| T2 | 2·00 (0·97) | 2·12 (0·89) | -0·12 | -0·76 | 176 | ·446 | -0·14 |
| T3 | 1·71 (1·17) | 2·32 (0·97) | -0·60 | -3·36 | 186 | ·001 | -0·59 |
| T4 | 1·74 (0·94) | 2·08 (0·98) | -0·34 | -1·99 | 186 | ·048 | -0·35 |
| T5 | 1·36 (0·98) | 2·15 (0·93) | -0·79 | -4·79 | 186 | ·000 | -0·84 |
| T6 | 1·57 (0·99) | 2·05 (0·88) | -0·48 | -3·00 | 186 | ·003 | -0·53 |
| T7 | 1·67 (1·18) | 2·12 (0·83) | -0·45 | -2·78 | 186 | ·006 | -0·49 |
| T8 | 1·67 (1·00) | 2·01 (0·82) | -0·34 | -2·25 | 185 | ·026 | -0·39 |
| T9 | 1·62 (1·01) | 1·98 (0·94) | -0·36 | -2·15 | 185 | ·033 | -0·38 |
| T10 | 1·71 (1·40) | 2·02 (0·84) | -0·31 | -1·78 | 179 | ·077 | -0·32 |
|  |  |  |  |  |  |  |  |

An independent samples *t* test indicated that the mean age of males (*M* = 19.96, *SD* = 0.93) did not significantly differ from that of females (*M* = 19.82, *SD* = 0.98), *t*(186) = 0.92, *p* = .361, Cohen’s *d* = 0.16.
